# Diagnostic Applications of Point‑of‑Care Ultrasound in Emergency Medicine: A Narrative Review

**DOI:** 10.7759/cureus.101501

**Published:** 2026-01-14

**Authors:** Yash Sharma, Babajide Obidigbo, Naomi P

**Affiliations:** 1 Trauma and Orthopaedics, Royal Victoria Hospital, Belfast, GBR; 2 Orthopaedics and Trauma, London Northwest University Healthcare NHS Trust, London, GBR; 3 Obstetrics and Gynaecology, York University, York, GBR

**Keywords:** emergency medical service, emergency obstetric care, pocus integration, trauma imaging, ultrasound (u/s)

## Abstract

Point‑of‑care ultrasound (POCUS) has become an integral diagnostic tool in emergency medicine, providing rapid bedside imaging that supports timely clinical decision‑making in acutely ill and injured patients. This narrative review examines the diagnostic applications of POCUS in emergency department settings, with emphasis on trauma assessment, lung ultrasound, focused cardiac ultrasound, evaluation of abdominal aortic aneurysm, and early pregnancy complications. The literature review was conducted using PubMed to identify peer‑reviewed studies relevant to diagnostic POCUS use in emergency medicine. The reviewed evidence demonstrates that POCUS improves diagnostic accuracy, accelerates time to critical interventions, and enhances patient triage across a broad range of emergency presentations. Key diagnostic applications include the focused assessment with sonography in trauma (FAST) and extended FAST examinations, evaluation of acute respiratory and cardiac conditions, rapid bedside identification of abdominal aortic aneurysm, and early detection of ectopic pregnancy. In summary, diagnostic POCUS represents a versatile, efficient, and clinically impactful modality that continues to enhance the delivery of emergency care.

## Introduction and background

Point-of-care ultrasound (POCUS) has become a core diagnostic modality in modern emergency medicine, enabling rapid bedside imaging that complements clinical assessment and supports early decision-making in time-critical situations [1-3]. Advances in ultrasound technology, including improved image quality, increased portability, and reduced cost, have facilitated widespread adoption of POCUS across emergency departments worldwide [1,2]. As a result, emergency physicians increasingly rely on POCUS to evaluate patients presenting with trauma, respiratory distress, circulatory shock, abdominal pain, and early pregnancy complications [1-4].

The diagnostic value of POCUS lies in its ability to provide immediate, dynamic information that can be directly integrated into bedside evaluation. Unlike traditional imaging modalities, POCUS can be performed repeatedly, does not expose patients to ionizing radiation, and often reduces delays associated with patient transport or radiology availability [3-5]. Multiple studies have demonstrated that emergency physician-performed ultrasound improves diagnostic accuracy, shortens time to intervention, and enhances patient triage, particularly in critically ill or hemodynamically unstable patients [1,6].

POCUS has well‑established applications across multiple diagnostic domains in emergency medicine. In trauma care, the focused assessment with sonography in trauma (FAST) examination allows rapid detection of intraperitoneal and pericardial fluid, while the extended FAST (eFAST) examination further enhances diagnostic capability through evaluation of pneumothorax and hemothorax [2,3,7]. In patients with acute respiratory symptoms, lung ultrasound facilitates prompt identification of pneumothorax, pleural effusion, pulmonary edema, and consolidation, often with greater sensitivity than conventional chest radiography [5,8-11]. Focused cardiac ultrasound provides immediate assessment of ventricular function and pericardial effusion, supporting early identification of reversible causes of shock [6,12,13].

Additional diagnostic applications include evaluation of the abdominal aorta for suspected abdominal aortic aneurysm and early pregnancy assessment to rapidly differentiate intrauterine pregnancy from ectopic pregnancy. In these contexts, timely bedside ultrasound can significantly reduce delays to definitive management and improve patient outcomes [14-17]. Given the expanding evidence base supporting its diagnostic accuracy and clinical utility, POCUS is increasingly regarded as an extension of the physical examination rather than a replacement for comprehensive imaging [1,4].

This narrative review aims to provide a focused overview of the diagnostic applications of POCUS in emergency medicine. By summarizing current evidence across major clinical domains, this review highlights the role of POCUS in enhancing diagnostic efficiency, improving workflow, and supporting high‑quality, patient‑centered emergency care [1-4].

Methods

A narrative literature review was conducted in May 2025 to identify studies evaluating the diagnostic applications of point‑of‑care ultrasound (POCUS) in emergency medicine. PubMed was searched for articles published from database inception to May 2025 using keywords including “point‑of‑care ultrasound,” “emergency medicine ultrasound,” “FAST exam,” “eFAST,” “lung ultrasound,” “focused cardiac ultrasound,” “abdominal aortic aneurysm ultrasound,” and “ectopic pregnancy ultrasound.”

Studies were screened for relevance to the diagnostic use of POCUS in emergency department settings. The eligible publications included original research articles, observational studies, systematic reviews, meta‑analyses, and narrative reviews published in peer‑reviewed, PubMed‑indexed journals with verifiable digital object identifiers (DOIs). Studies focusing exclusively on procedural ultrasound applications or non‑emergency clinical settings were excluded.

A total of 31 articles were identified during the initial literature search, of which 24 studies met the inclusion criteria and were included in the final narrative synthesis. Additional relevant studies were identified through manual review of the reference lists of key publications to ensure comprehensive coverage of foundational and high‑impact literature. As this study was conducted as a narrative review, formal systematic review protocols and quality assessment tools were not employed.

## Review

Diagnostic applications of POCUS in emergency medicine

POCUS has become an essential diagnostic adjunct in emergency medicine due to its ability to provide immediate, bedside imaging that can be integrated directly into clinical assessment. Unlike traditional imaging modalities, POCUS allows dynamic and repeatable examinations, facilitating rapid diagnostic clarification in critically ill or unstable patients. Its portability, absence of ionizing radiation, and growing evidence base have contributed to its widespread adoption across emergency departments worldwide [1,4,18].

Trauma evaluation (FAST examination)

The FAST examination is one of the earliest and most established diagnostic applications of POCUS in emergency medicine. FAST is primarily used to detect free fluid in the peritoneal, pericardial, and pleural spaces, serving as a rapid screening tool for internal hemorrhage in trauma patients. Meta‑analyses have demonstrated that FAST has high specificity for identifying clinically significant intraperitoneal bleeding, particularly in hemodynamically unstable patients, where rapid decision‑making is critical [2,3].

The eFAST examination builds upon the traditional FAST protocol by incorporating lung ultrasound to evaluate for pneumothorax and hemothorax. This extension significantly enhances diagnostic capability in trauma settings, particularly when chest radiography or computed tomography is delayed or unavailable. Systematic reviews and meta‑analyses have shown that eFAST has high specificity for detecting traumatic pneumothorax and can expedite life‑saving interventions in unstable patients [2,5]. A summary of major trauma‑related diagnostic applications is provided in Table 1.

**Table 1:** Major diagnostic applications of point‑of‑care ultrasound (POCUS) in emergency medicine POCUS: point‑of‑care ultrasound; FAST: Focused Assessment with Sonography in Trauma; eFAST: extended Focused Assessment with Sonography in Trauma; FoCUS: focused cardiac ultrasound; CT: computed tomography.

Diagnostic Domain	Primary Clinical Use	Key Diagnostic Value	Supporting References
Trauma (FAST)	Detection of intraperitoneal and pericardial free fluid	Rapid identification of internal hemorrhage in unstable trauma patients	2, 3
Trauma (eFAST)	Detection of pneumothorax and hemothorax	Improves diagnostic accuracy when CT or radiography is delayed	2, 5
Lung ultrasound	Pneumothorax, pleural effusion, pulmonary edema, consolidation	Higher sensitivity than chest radiography for several acute conditions	5, 7–10
Focused cardiac ultrasound (FoCUS)	Ventricular function, pericardial effusion, shock assessment	Early identification of reversible causes of shock	1, 12, 13
Abdominal aorta	Identification of abdominal aortic aneurysm	Rapid bedside detection of aneurysmal dilation	14, 15
Early pregnancy	Intrauterine vs ectopic pregnancy differentiation	Reduces time to diagnosis and intervention	16, 17

Lung ultrasound

Lung ultrasound is a highly effective diagnostic modality for evaluating acute respiratory conditions in the emergency department. It is commonly used to identify pneumothorax, pleural effusion, pulmonary edema, and lung consolidation. Multiple meta‑analyses have demonstrated that lung ultrasound is more sensitive than chest radiography for the detection of pneumothorax and pleural effusion, particularly in critically ill and trauma patients [5,7,10].

In patients presenting with undifferentiated dyspnea, POCUS enables rapid differentiation between cardiogenic and non‑cardiogenic causes of respiratory distress. The presence of bilateral B‑lines supports pulmonary edema, while focal consolidations and pleural abnormalities suggest pneumonia or other parenchymal disease. Large systematic reviews have confirmed high diagnostic accuracy of lung ultrasound for cardiopulmonary causes of dyspnea and acute pulmonary edema, supporting its role as a first‑line imaging modality in emergency settings [6,8,9]. These findings highlight the value of lung ultrasound as a rapid, accurate, and radiation‑free diagnostic tool for acute respiratory presentations [5-10].

Cardiac ultrasound

Focused cardiac ultrasound (FoCUS) plays a critical role in the emergency evaluation of patients with shock, chest pain, and cardiac arrest. It allows rapid bedside assessment of global ventricular function, pericardial effusion, right ventricular dilation, and intravascular volume status. When incorporated into structured protocols such as the rapid ultrasound in shock (RUSH) examination, FoCUS improves diagnostic accuracy in patients with undifferentiated hypotension [1].

Several studies have demonstrated good agreement between emergency physician-performed FoCUS and formal echocardiography for the identification of major cardiac pathology. FoCUS has been shown to reliably detect pericardial effusion, gross systolic dysfunction, and signs of right heart strain, enabling early recognition of potentially reversible causes of shock such as cardiac tamponade or massive pulmonary embolism [12,13]. Although FoCUS does not replace comprehensive echocardiography, its diagnostic utility in time‑critical emergency settings is well supported.

Abdominal aortic aneurysm (AAA) assessment

Abdominal aortic aneurysm (AAA) is a life‑threatening condition that may present with nonspecific abdominal, back, or flank pain. POCUS allows rapid bedside visualization of the abdominal aorta and has become a recommended first‑line imaging modality for suspected AAA in the emergency department. A systematic review demonstrated high sensitivity and specificity of emergency physician-performed ultrasound for detecting aneurysmal dilation, particularly for aneurysms greater than 3 cm in diameter [14].

Emergency ultrasound has also been shown to significantly reduce time to diagnosis and surgical consultation in patients with symptomatic or ruptured AAA. While imaging may be limited by factors such as obesity or bowel gas, POCUS remains a highly effective screening and triage tool and plays a crucial role in early recognition of this high‑mortality condition [14,15].

Early pregnancy and gynecologic emergencies

POCUS is a cornerstone of early pregnancy evaluation in emergency medicine, particularly in patients presenting with abdominal pain or vaginal bleeding. Bedside ultrasound enables rapid differentiation between intrauterine pregnancy and ectopic pregnancy, which is essential in hemodynamically unstable patients. Meta‑analyses have demonstrated that emergency physician-performed ultrasound has high diagnostic accuracy for identifying ectopic pregnancy and significantly reduces delays to diagnosis and management [16].

In addition to improved diagnostic accuracy, POCUS has been associated with shorter emergency department length of stay and more rapid initiation of definitive care compared with reliance on radiology-performed ultrasound alone [11]. These benefits underscore the importance of bedside ultrasound as a safe, efficient, and clinically impactful diagnostic tool in early pregnancy emergencies [16,17]. A consolidated overview of key diagnostic applications is presented in Table 1, while Table 2 summarizes common diagnostic strengths and limitations across major POCUS applications.

**Table 2:** Diagnostic strengths and limitations of point‑of‑care ultrasound (POCUS) across major emergency medicine applications. POCUS: point‑of‑care ultrasound; FAST: Focused Assessment with Sonography in Trauma; eFAST: extended Focused Assessment with Sonography in Trauma; FoCUS: focused cardiac ultrasound; AAA: abdominal aortic aneurysm; ED: emergency department.

Diagnostic Area	Key Strengths	Common Limitations	Supporting References
FAST/eFAST	Rapid bedside assessment; repeatable; high specificity in unstable patients	Limited sensitivity for small or retroperitoneal injuries; operator dependent	[2,3,5]
Lung ultrasound	High diagnostic accuracy; radiation‑free; rapid assessment of dyspnea	Image artifacts; reduced accuracy in obesity or subcutaneous emphysema	[5,7–10]
FoCUS	Immediate cardiac assessment; guides shock management	Does not replace formal echocardiography; image quality varies	[1,12,13]
AAA evaluation	Fast bedside screening; reduces time to surgical consultation	Limited by bowel gas or body habitus	[14,15]
Early pregnancy ultrasound	Rapid differentiation of ectopic pregnancy; improves ED flow	Very early gestation may yield indeterminate findings	[16,17]

Discussion

The integration of POCUS into emergency medicine represents a significant shift toward rapid, bedside diagnostic strategies that enhance clinical efficiency and patient outcomes. The evidence summarized in this review demonstrates that diagnostic POCUS provides timely and actionable information across a wide range of emergency presentations, supporting early clinical decision‑making in time‑critical situations. Its clinical impact is particularly evident in trauma, acute respiratory distress, circulatory shock, abdominal emergencies, and early pregnancy complications, where delays in diagnosis may significantly affect morbidity and mortality [1,4,18]. Real-world observational studies from community emergency departments have demonstrated increasing utilization of POCUS, supporting its role in routine diagnostic workflows beyond academic centers [19].

One of the principal strengths of POCUS is its ability to deliver real‑time imaging that can be immediately correlated with clinical findings at the bedside. In trauma care, the FAST and eFAST examinations enable rapid identification of intraperitoneal bleeding and traumatic pneumothorax, guiding early operative or interventional management in hemodynamically unstable patients [2,3,5]. Recent investigations have further confirmed the diagnostic accuracy of POCUS in high‑severity trauma, reinforcing its value in early risk stratification and time‑critical clinical decision‑making [20]. Similarly, lung ultrasound has demonstrated high diagnostic accuracy for pneumothorax, pleural effusion, and pulmonary edema, frequently outperforming chest radiography and allowing clinicians to rapidly differentiate causes of acute dyspnea in the emergency department [5-10].

Focused cardiac ultrasound further expands the diagnostic utility of POCUS by enabling prompt assessment of ventricular function, pericardial effusion, and hemodynamic status. When incorporated into structured protocols such as the RUSH examination, focused cardiac ultrasound supports rapid bedside evaluation and facilitates early identification of potentially reversible etiologies of shock [1,12,13]. In abdominal emergencies, bedside ultrasound evaluation of the abdominal aorta has proven highly sensitive for detecting abdominal aortic aneurysm, reducing time to diagnosis and expediting surgical consultation in high‑risk patients [14,15]. In early pregnancy, emergency physician‑performed ultrasound plays a critical role in identifying ectopic pregnancy and minimizing delays to definitive care, contributing to improved patient safety and emergency department flow [16,17].

Despite these advantages, the diagnostic performance of POCUS is influenced by operator experience, training, and patient‑specific factors. Variability in image acquisition and interpretation remains a recognized limitation, particularly in patients with obesity, subcutaneous emphysema, or challenging anatomy. In addition, while POCUS excels as a rapid screening and decision‑support tool, it does not replace comprehensive imaging or specialist‑performed studies when further diagnostic clarification is required. A summary of key diagnostic strengths and common limitations across major POCUS applications is presented in Table 2.

Broad narrative reviews and implementation studies have highlighted the expanding scope of POCUS in emergency medicine, emphasizing its impact on diagnostic efficiency, clinician confidence, and integration into standard emergency care pathways [21,22]. Evidence in undifferentiated shock further supports the diagnostic contribution of bedside ultrasound protocols in early classification of shock states and time‑critical decision‑making [23]. Additional implementation‑focused analyses of emergency department imaging utilization also support the growing role of POCUS as a frontline diagnostic modality in contemporary emergency care [24].

Limitations

This narrative review has several limitations. As this was a narrative rather than a systematic review, study selection may be subject to selection bias, publication bias, outcome reporting bias, confirmation bias, and interpretive bias. In addition, not all relevant publications may have been identified, and the included literature may emphasize studies reporting positive findings that support the proposed narrative regarding the clinical utility of point‑of‑care ultrasound. The included literature encompasses studies with varying designs, sample sizes, and clinical settings, which may introduce heterogeneity in reported outcomes. Additionally, the diagnostic performance of point‑of‑care ultrasound is influenced by operator experience, training, and patient‑specific factors, potentially limiting generalizability across all emergency care environments. Despite these limitations, the reviewed evidence consistently supports the diagnostic value of POCUS in emergency medicine.

## Conclusions

Point‑of‑care ultrasound has become an indispensable diagnostic modality in contemporary emergency medicine, offering rapid, bedside imaging that enhances diagnostic accuracy and supports timely clinical decision‑making. Across trauma, cardiopulmonary, abdominal, and early pregnancy presentations, POCUS consistently improves patient triage, reduces time to critical intervention, and integrates seamlessly with clinical assessment in high‑acuity settings. As ultrasound technology continues to advance and training becomes more standardized, the role of diagnostic POCUS is expected to further expand, reinforcing its importance in delivering efficient, high‑quality, patient‑centered emergency care.
